# Lost for emotion words: What motor and limbic brain activity reveals about autism and semantic theory

**DOI:** 10.1016/j.neuroimage.2014.09.046

**Published:** 2015-01-01

**Authors:** Rachel L. Moseley, Yury Shtyrov, Bettina Mohr, Michael V. Lombardo, Simon Baron-Cohen, Friedemann Pulvermüller

**Affiliations:** a MRC Cognition and Brain Sciences Unit, Cambridge, UK; b Autism Research Centre, Department of Psychiatry, University of Cambridge, UK; c Centre for Functionally Integrative Neuroscience, Aarhus University, Denmark; d Centre for Cognition & Decision Making, Faculty of Psychology, Higher School of Economics, Moscow, Russia; e Charité Universitätsmedizin, Department of Psychiatry and Psychotherapy, Campus Benjamin Franklin, Berlin, Germany; f Department of Psychology, University of Cyprus, Cyprus; g Cambridgeshire and Peterborough NHS Foundation Trust, CLASS Clinic, UK; h Brain Language Laboratory, Department of Philosophy and Humanities, Freie Universität Berlin, Germany; i Center for Applied Neuroscience, University of Cyprus, Cyprus

**Keywords:** Autism, Emotion, Embodied cognition, Semantics

## Abstract

Autism spectrum conditions (ASC) are characterised by deficits in understanding and expressing emotions and are frequently accompanied by alexithymia, a difficulty in understanding and expressing emotion words. Words are differentially represented in the brain according to their semantic category and these difficulties in ASC predict reduced activation to emotion-related words in limbic structures crucial for affective processing. Semantic theories view ‘emotion actions’ as critical for learning the semantic relationship between a word and the emotion it describes, such that emotion words typically activate the cortical motor systems involved in expressing emotion actions such as facial expressions. As ASC are also characterised by motor deficits and atypical brain structure and function in these regions, motor structures would also be expected to show reduced activation during emotion-semantic processing. Here we used event-related fMRI to compare passive processing of emotion words in comparison to abstract verbs and animal names in typically-developing controls and individuals with ASC. Relatively reduced brain activation in ASC for emotion words, but not matched control words, was found in motor areas and cingulate cortex specifically. The degree of activation evoked by emotion words in the motor system was also associated with the extent of autistic traits as revealed by the Autism Spectrum Quotient. We suggest that hypoactivation of motor and limbic regions for emotion word processing may underlie difficulties in processing emotional language in ASC. The role that sensorimotor systems and their connections might play in the affective and social-communication difficulties in ASC is discussed.

## Highlights

• Motor and limbic cortices are typically activated by emotion words.
• In ASC, activity in these key regions is reduced for this word type specifically.
• Hypoactivity does not appear in brain regions unrelated to emotion word processing.
• ASC motor systems' hypoactivity to emotion words correlates with symptomatology.
• Motor deficits may help explain emotion processing problems and other ASC symptoms.

## Introduction

Inherent in Kanner's first description of autism as a ‘disturbance of affective contact’ (Kanner, 1943), the domain of emotion has been cardinal throughout the history of autism research. Disturbances in the affective domain may help to explain why individuals with autism spectrum conditions (ASC) have difficulty in connecting, socializing, communicating, and understanding the hidden mental world of others that drives much of social behaviour (Baron-Cohen, 1995). A recent, methodologically rigorous meta-analysis of the emotion recognition literature in autism suggests that a pervasive deficit exists (Uljarevic and Hamilton, 2013), extending to understanding emotions in vocal cues and nonverbal gestures (Braverman et al., 1989, Hobson, 1986a, Hobson, 1986b, Rutherford et al., 2002, Golan et al., 2007, Humphreys et al., 2007, Philip et al., 2010). In terms of emotional expression, studies in autism also indicate lower responsivity to emotional displays of others (Sigman et al., 1992, Kasari et al., 1993), a lack of spontaneous mimicry of others' facial expressions (McIntosh et al., 2006, Beall et al., 2008, Oberman et al., 2009), and attenuated physiological response to emotional expressions, pain and distress in others (Corona et al., 1998, Ben Shalom et al., 2006, Bölte et al., 2008, Minio-Paluello et al., 2009). Vocalisations and facial expressions of affect in autism are characteristically flat or neutral (Snow et al., 1987, Yirmiya et al., 1989, Capps et al., 1993), and may be inappropriately disconnected from the social context in which they appear (Neuman and Hill, 1978, Dawson and McKissick, 1984, Hobson et al., 2006). Finally, difficulty in identifying and describing emotions verbally, known as alexithymia, is much more prevalent in people with ASC (Lombardo et al., 2007, Hill et al., 2004) and their parents (Szatmari et al., 2008). In summary, it seems that individuals with ASC are atypical in how they express emotions in comparison to typically-developing peers and that, likewise, their perception and mirroring of emotions are reduced, if not impaired.

This pattern of emotion expression and perception deficits is one component of the difficulty in mentalising, the ability to represent one's own emotional states and thought processes and those of others (Happé, 1994, Baron-Cohen, 1995, Lombardo et al., 2007, Silani et al., 2008, White et al., 2009, Lombardo and Baron-Cohen, 2011). In this sense, deficits in self-referential processing may alter an individual's ability to use the self as a proxy for simulating the mental lives of others. A deficit in representation and/or recognition of one's own emotions would impair attempts to accurately simulate others via oneself (Lombardo and Baron-Cohen, 2011).

The more generalized role of simulation in mental operations is increasingly recognised in the field of cognitive neuroscience, where Barsalou (2008) names it “a core form of computation in the brain” (pp. 618–619). A proposal which has recently gained speed with much empirical support is that self-performed actions (including those involved in emotional expression) and the perceptual consequences of the same lead to linked action–perception representations that are later used in cognitive processing and social interaction (Pulvermüller, 2013, Pulvermüller et al., 2014). In action cognition, for example, researchers have suggested that action goals are simulated in the motor systems of observers in order to understand the intentions underlying actions, such as whether or not the actor intends to eat the object (Cattaneo et al., 2007). In the context of the mirror neuron theory, these joint action–perception circuits, consisting of these self-same sensorimotor neurons with visual and/or auditory properties, have been found underactive in autism (Williams et al., 2001, Cattaneo et al., 2007, Rizzolatti and Fabbri-Destro, 2010, Moseley et al., 2013a).

Given that alexithymia in autism is a *linguistic* deficit in processing the emotions of self and others, this implies that there is atypical processing of words semantically related to emotions. Prior work in autism suggests that these individuals do indeed show difficulty in understanding and using emotional and cognitive mental state terms (such as “dread”, “thought”: see Hobson and Lee, 1989, Capps et al., 1992, Tager‐Flusberg, 1992, Baron-Cohen et al., 1994, Baron‐Cohen et al., 1986, Happé, 1994, Tager-Flusberg and Sullivan, 1994, Tager-Flusberg and Sullivan, 1995, Jolliffe and Baron-Cohen, 1999) as well as an inability to link mental state terms to emotional information present in features of the eyes (Baron-Cohen et al., 1997, Baron-Cohen et al., 2001). The neural correlates of this deficit are, however, unknown. In the present study we used event-related fMRI to investigate brain systems activated when people with ASC process abstract emotion words. Our hypotheses about atypical cortical activity during emotion word processing focused on two key areas.

In typically developing (TD) individuals, understanding the meaning of action and emotion words and concepts seems to involve the cortical motor system (Pulvermüller and Fadiga, 2010, Moseley et al., 2012). Our prior work has shown that individuals with autism show hypoactivity of cortical motor systems when they process action words and concepts (Moseley et al., 2013a, Moseley et al., 2013c), and this is consistent with atypical structure of the motor cortex (Mostofsky et al., 2007) and movement impairments in ASC (see Fournier et al., 2010, for review). The motor regions unexpectedly inactive during action word processing in ASC were the same as those found particularly active when TD individuals processed abstract emotion words (Moseley et al., 2012). Theoretically, this ‘motor embodiment’ of emotion words suggests that the link between an emotion word and the emotional state it expresses depends on emotion expression in action (Wittgenstein, 1953, Pulvermüller, 2012, Pulvermüller, 2013). In early language acquisition, emotion expression by infants provides a natural context for teaching emotion words and, therefore, the motor and limbic regions for emotion expression may be woven into the semantic representations of abstract emotion-related words. As TMS and work in brain-damaged patients shows that somatosensory and motor regions along with limbic emotion processing areas in insular cortex are necessary for the perception of emotion-related information immanent to the face (Pitcher et al., 2008, Adolphs et al., 2000), and these same areas are also active in emotion word processing (Moseley et al., 2012, Vigliocco et al., 2013), we hypothesised that these cortical motor and limbic systems would be affected in autism during emotion word processing and might reflect the degree of autistic traits in ASC.

An additional hypothesis focuses on limbic areas involved in emotion processing (Calder et al., 2001). A range of these regions, including orbitofrontal and frontopolar cortex, anterior cingulate gyrus, insula, and basal ganglia (putamen, caudate, and globus pallidum), are involved in emotion *word* processing (for review see Moseley et al., 2012, Vigliocco et al., 2013). Because these regions are specifically activated by *emotion-related* language, this subset of limbic areas, along with motor systems, provides a putative cortical basis for ‘simulation’ of word meaning and affective semantics more generally. It has been suggested that, at the neurobiological level, strong emotional-affective associations of emotion words are mechanistically organised as ‘limbic tails’ of cortical cell assemblies reaching into subcortical structures of the limbic system (Pulvermüller and Schumann, 1994). In addition to the aforementioned abnormalities of cortical motor systems, people with ASC also show atypical activity and structure in many of these limbic regions (Bauman and Kemper, 1994, Raymond et al., 1995, Haznedar et al., 1997, Haznedar et al., 2000, Aylward et al., 1999, Howard et al., 2000, Ohnishi et al., 2000, Salmond et al., 2003, Barnea-Goraly et al., 2004, Schumann et al., 2004, Schumann and Amaral, 2006, Girgis et al., 2007, Bonilha et al., 2008, Cheung et al., 2009, McAlonan et al., 2009, Pugliese et al., 2009, Uddin and Menon, 2009), so we predict additional hypo-activity in limbic systems when people with ASC process emotion-related words.

In summary, activation of motor and limbic areas during abstract emotion word processing seen in TD individuals may be atypical in ASC, partly due to their deficits in emotion processing and thus limbic activation, and to deficits in emotion expression in action and thus motor system activity. Whereas limbic hypoactivation might be predicted by the common emotion processing deficits in ASC, the additional prediction of motor hypoactivity in emotion word processing rests on the semantic link between emotion words and motor systems (Moseley et al., 2012). If the semantic link between an emotion and the word denoting it is via emotion expression in motor behaviour, the motor difficulties reported in ASC imply that this link will be atypical even during single word reading and comprehension, a task unrelated to overt emotion processing. Therefore, if words denoting abstract emotional states draw on cortical motor and limbic regions during processing, atypical functioning may be apparent in both of these regions when individuals with ASC simply read these words. In comparison with words denoting animals or abstract verbs, neither of which are especially linked with motor or limbic regions, we predicted that individuals with ASC would show a category-specific abnormality during emotion word processing that should be specific to the motor and limbic areas that are atypical in ASC and associated with emotion word processing in typical controls.

## Materials and methods

### Participants

Right-handed, native English-speaking participants comprising 18 high-functioning adults with an ASC (mean age: 30.4 years [standard deviation (SD): 10]; mean IQ: 113.5 [SD: 23]) and 18 age- and IQ-matched typically-developing (TD) controls (mean age: 28.6 years [SD: 11.7]; mean IQ: 110.2 [SD: 12.3]). Data from TD participants were previously published in Moseley et al. (2012); here, a new and independent analysis compares these participants and individuals with ASC who were recruited from the volunteer database at www.autismresearchcentre.com, hosted by the Autism Research Centre at Cambridge University, UK. All ASC participants had been previously clinically diagnosed using DSM-IV criteria: 17 met criteria for Asperger Syndrome, one for PDD-NOS (pervasive developmental disorder not otherwise specified). The ASC group scored significantly higher than the control group (t [32] = 6.857, p < .001) on the Autism Spectrum Quotient (AQ: Baron-Cohen et al. 2001b), with a mean score of 34 (SD: 10) in comparison to the control mean AQ of 13 (SD: 5). All but 4 of the ASC group scored above 26 on this test, a cut-off point that captures the majority of adults with autism (Woodbury-Smith et al., 2005). In order to keep the procedure short, this experiment matched participants only in full-scale IQ (Cattell and Cattell, 1960). Although they were not directly matched in verbal IQ, both had performed with no significant differences in accuracy in a general language processing task reported in a previous experiment (Moseley et al., 2013a) and the participants with ASC were high-functioning individuals who had or were currently working or studying, and so seemed to be comparable in their ability to read and understand the task.

All participants gave written informed consent prior to participating in this study and were remunerated for their time. Ethics approval was provided by NHS Research Ethics Service Cambridgeshire.

### Stimuli

A semantic rating study was carried out prior to the fMRI experiment in order to assess semantic properties for a large corpus of words: these included arousal, valence, imageability, concreteness, visual-relatedness, form-relatedness, colour-relatedness, and action-relatedness. Arousal and valence ratings have previously been employed in work on emotional-affective meaning (Osgood et al., 1975, Bradley and Lang, 1994) to classify words as emotional or non-emotional. Our previous work (Moseley et al., 2012) used an additional explicit rating of emotion-relatedness to identify words (primarily verbs) specifically used to speak about emotions, such as “dread”, “hate” and “fear”. As words with action-related meanings (e.g. “kick”) are also known to activate cortical motor systems, we excluded more concrete emotional items with sensorimotor associations used in the previous study (such as “wail” or “scream”), which were less optimal for exploring ‘pure’ abstract emotion concepts (see Moseley et al., 2012 for details of this and the rating procedure). As such, a more refined category of the most abstract emotion words (20) was contrasted with 40 animal names (nouns such as “hawk”, “mouse”, “sheep”) and 40 abstract verbs (e.g. “heal”, “dwell”, “waive”, which also lacked overt or concrete sensorimotor associations with the body). Words were matched in length, number of neighbours, word frequency, bigram and trigram frequency (see Table S1 in supplementary information for psycholinguistic and semantic properties of all experimental word categories). In order to disguise the focus of the study, the experimental words were dispersed among 240 filler words and 120 hash-mark strings (###) which, matched in length with the 360 word stimuli, were used as a low-level visual baseline unrelated to language.

Animal names were included as a word category unrelated to actions, in order to rule out the possibility of indiscriminate motor activity during general language processing. Whilst a lexical confound existed with the animal name category (nouns), both the emotion and abstract word categories were verbs (or had strong primary use as verbs): any atypical activity which appeared for emotion words but not for abstract verbs could not therefore be ascribed to a general problem with this lexical category. Though matched in word frequency, animal words were rated as significantly more familiar than our other experimental word categories. Abstract verbs, which were not significantly different to emotion words in familiarity (t [58] = .246, p = .807) or the concreteness of their meaning (t [58] = .510, p = .612), were the stronger control category. All three experimental word categories can be seen in Table S2 (Supplementary materials): they were presented in lowercase just as they appear.

### Procedure and experimental design

Prior to scanning, all subjects completed Form A of the Cattell Culture Fair test (Cattell and Cattell, 1960) and the Autism Spectrum Quotient (AQ: Baron‐Cohen et al., 2001b), in order to obtain measures of IQ and number of autistic traits. The Cattell Culture Fair test is a non-verbal IQ test frequently employed in cognitive neuroscience (Duncan et al., 2000). It is believed to provide the strongest measure of “fluid” intelligence (Cattell, 1980, Jensen, 1980, Colom and García-López, 2002, Colom et al., 2003), that which is distinct from learned verbal material (which influences some subtests of the Wechsler Adult Intelligence Scale [Wechsler, 1944]) and which is considered to reflect “true” problem-solving or abstract reasoning ability (Carroll, 1993, Gustafsson, 1984) rather than ‘crystallised’ intelligence (learned knowledge).

Event-related fMRI was used to compare brain activity during passive silent reading of experimental words, which were presented tachistoscopically for 150 ms. This short presentation time was employed to discourage saccades and encourage continuous attention in order to perform well on the task. Participants were asked to focus on a central fixation cross (presented for an average of 2350 ms) following the presentation of word stimuli, with SOA varied at an average of 2500 ms. Two pseudo-randomised stimulus lists were counterbalanced between subjects, who were instructed to read the words silently without moving their lips or tongue. Observation during scanning ruled out the effect of overt movements on results.

Immediately following scanning, an unseen word recollection test containing a combination of experimental and novel distracter words asked participants to rate how confident they were that each word had appeared in the experimental task. Accurate results confirmed that participants had maintained attention on the experimental task. T-tests confirmed that both groups performed above chance (average hit rate: controls = 76.2% (SD = 18.1%), ASC = 76.2% (SD: 19.1%)), with no significant difference appearing between them in the number of correct answers (t [34] = − .018, p = .913).

### Imaging analysis

Participants were scanned in a 3 T Tim-Trio scanner with a 12-channel head-coil attached. Functional scans consisted of 32 slices covering the whole brain in descending order (slice thickness: 3 mm, in-plane resolution: 3 × 3 mm, inter-slice distance: 0.75 mm), and echo-planar sequence parameters were TR = 2000 ms TE = 30 ms and flip angle = 78°. SPM5 (Wellcome Department of Imaging Neuroscience, London, UK) was employed for all processing stages, including slice-timing and re-aligning using sinc interpolation, co-registration of images to structural T1 images and normalisation of the previous to the 152 subject T1 template of the Montreal Neurological Institute (MNI). Transformation parameters were applied to co-registered EPI images, which were also resampled with a spatial resolution of 2 × 2 × 2 mm and spatially smoothed with an 8-mm full-width half-maximum Gaussian kernel.

Single-subject, second level and group statistical contrasts were computed using the canonical haemodynamic response function (HRF) of the general linear model. Low-frequency noise was removed by applying a high-pass filter of 128 s. Onset times for each stimulus were extracted from E-prime output files and integrated into a model for each block in which each stimulus category was modelled as a separate event. Group data were then analysed with a random-effects analysis. Direct statistical contrasts between groups for each word category were computed and voxel coordinates reported in MNI standard space. Statistical thresholding was initially set at a liberal threshold of p < .001, uncorrected, in order to observe the activation landscape evoked by words but was then followed up by more stringent thresholding at a Family-Wise Error (FWE) corrected level of p < .05.

In addition to whole-brain analysis, a regions-of-interest (ROI) investigation was undertaken, using the MarsBar function of SPM5, in order to look for statistical interactions between brain regions, word category and group in an ANOVA approach. ROIs were created by pooling participants from both groups and assessing regions where brain activity was robust for the general language contrast of all words against the hash-mark baseline. The three clusters (each with 3 peak loci) with most highly significant t values at a FWE-corrected threshold of p < .05 were selected for detailed analysis in 5 mm-radius ROIs. Activation was collapsed across the 3 peaks within each cluster to form the factor of Area for statistical analysis. These three Areas for analysis therefore included a left-hemispheric temporal cluster (its collapsed peaks located in inferior temporal [− 60, − 34, − 2] and fusiform gyrus [− 40, − 44, − 18, and − 42, − 4, − 44]: henceforth referred to as the “Temporal cluster”); a left-hemispheric cingulate cluster (collapsed loci in dorsal medial BA 6 [− 6, − 2, 64] and cingulate cortex at BA 32 [− 6, 16, 40] and BA 23 [− 8, − 8, 46]; henceforth the “Cingulate cluster”); and a right hemispheric motor cluster (its collapsed loci in primary motor [BA 4: 52, − 8, 42] and premotor cortex [BA 6: 62, 4, 12, and 60, 2, 38]; henceforth the “Motor cluster”). As can be seen in Fig. 2 Part A, the activity evoked by general reading spread across disparate areas, and consequently, a peak arose within the same cluster as the temporal ROIs that was actually located in left premotor cortex (BA 6: − 42, − 4, 44). As this peak could not be included in the Temporal cluster within the ANOVA described below, it was analysed separately. After analysing at these 3 Areas (collapsed across 3 ROIs in each), their homologues in the contralateral hemisphere were computed, and these homologues (themselves collapsed from individual peaks into Areas mirroring those in the left hemisphere) were averaged with the originals in order to carry out a bilateral analysis.

Finally, correlations between AQ scores and activity in these three bilateral Areas for each of the three word categories (3 × 3 tests) were examined in the ASC group. On observing the data, two clear outliers were observed who scored very low in ASC traits, more than 10 points below the typical cut-off point of 26 (Woodbury-Smith et al., 2005). The AQ is not a diagnostic measure but in this previous work, it reliably identified people with ASC in 83% of tested cases. It was noted that one of our outliers was in fact the one participant with PDD-NOS, which has also been known as “atypical autism”, being a diagnostic label for cases which meet many but not all the prototypical symptoms of autism or which have additional, uncharacteristic symptoms. These two individuals were more comparable with the control group for AQ scores (see Supplementary materials, image S4). These two participants were identified as outliers in the ASC group on two fronts, being more than 2 standard deviations from the group's mean AQ and secondly through calculation of the median absolute deviation (see Leys et al., 2013, for details of this recommended method for outlier identification). They were removed from the correlation analysis but kept in brain-imaging analyses for the sake of matching group size; checks were made to guarantee that removal of these outliers did not affect significance in the other findings.

## Results

Activity during general word processing (all word categories against the hash-mark baseline), pooled across all participants, revealed widespread activity which was strongly left-lateralised across perisylvian language regions (fusiform to superior temporal cortex, inferior frontal gyrus), plus activity in the motor system bilaterally.

**Fig. 1:**
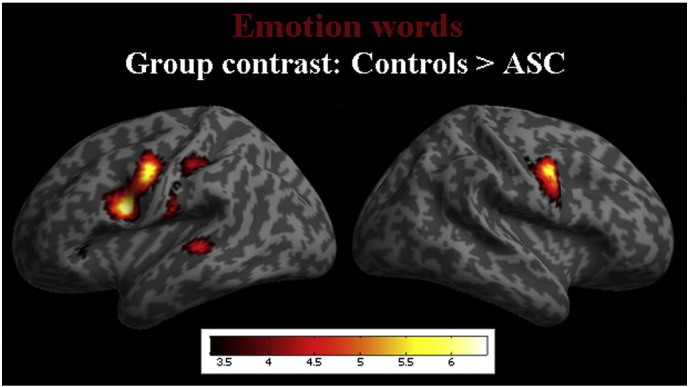
Statistical group contrast (controls > ASC) for emotion words (red). Activation is thresholded at p < .001, but the yellow parts of the activation clusters reflect activity which survived FWE (p < .05) correction.

Direct between-group statistical contrasts at a FWE-corrected whole-brain level revealed a range of cortical regions for each word category which were activated more strongly in the typically-developing (TD) control participants than in the ASC group. Regions where emotion words evoked greater activity in TD than ASC participants are displayed in Table 1 and Fig. 1 (p < 0.05, FWE corr.). These included a large cluster of voxels (n = 2438) encompassing left-hemispheric motor systems (BA 4 and 6) and the insula, and additional clusters in the left fusiform gyrus and the right-hemispheric motor system (BA 6). A left-hemispheric cluster including the anterior cingulate and caudate nucleus was also more strongly activated by emotion words in TD than ASC subjects, but this cluster did not survive FWE correction. Areas more strongly activated by the control word categories in the control participants are displayed in Supplementary Materials (Table S3), though none survived FWE correction. The contrast of ASC > control participants was non-significant at whole-brain level for each word category.

**Table 1:** Areas of greater activity for emotion words in Control vs. ASC participants.

	x y z	Cluster size	t	P (uncorr. 001)	P (FWE .05)
L. inferior and lateral sensorimotor cortex, dorsal premotor cortex (BA 6)	− 54, 4, 24	2438	6.40	.001	.002
L. insula	− 58, − 18, 22	699	6.11	.002	.009
L. inferior motor cortex (BA 4)	− 52, − 8, 44		5.98		.004
L. fusiform gyrus (BA 37)	− 42, − 42, − 12	447	6.09	.009	.013
L. fusiform gyrus (BA 37)	− 44, − 58, − 10		5.03		
R. BA 6	56, 0, 42	220	5.87	.051	.007
R. BA 6	50, − 14, 50	836	3.44	.001	
L. superior temporal (BA 22)	− 56, − 36, 2		4.91		
L. suppl. motor cortex (BA 6)	− 2, 4, 60		4.66		
L. caudate	− 8, 16, 18	92	4.01	.009	
L. anterior cingulate	− 4, 6, 28	62	3.88	.014	
R. anterior cingulate	10, 16, 28		4.45		
R. insula (BA 48)	30, 18, 14		4.10		

Table 1: MNI coordinates for the comparison of typically-developing (TD) controls vs. ASC groups (control > ASC) for emotion words. P values (cluster-level) are reported at an uncorrected (p < .001) and FWE-corrected (.05) level. Areas included in one row portray areas that arose as part of a cluster.

**Fig. 2:**
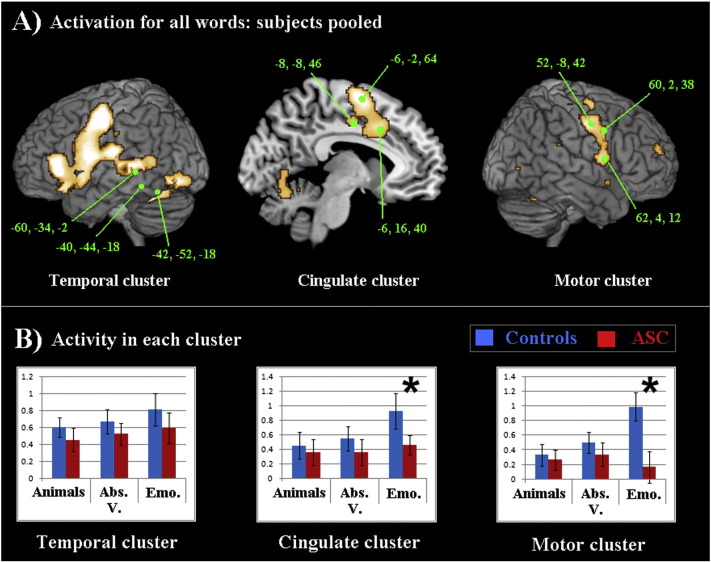
Part A): Activation for all words as contrasted against the hash-mark baseline, presented at a threshold of p < .001 for all subjects pooled. Individual loci, depicted above, were collapsed in the analysis to form one Temporal cluster, one Cingulate cluster and one Motor cluster. Part B): Graphs depict activity for each word category in each cluster. Significant Word Category × Group interactions, driven by between-group differences marked by asterisks (*), were found in the Cingulate and Motor clusters. Mean activation for the control participants is in blue, for the ASC participants in red. Error bars reflect standard error.

In a secondary ROI analysis, activation for each word category was examined in three large clusters of activity (left temporal, cingulate, and right motor cortex) that arose from the contrast of all words against the hash-mark baseline for all subjects pooled. The coordinates of the loci collapsed in each cluster can be seen in Fig. 2, part A.

These clusters were entered into an ANOVA including the factors Area (3 levels: Temporal, Cingulate, Motor), Word Category (3 levels: animal names, abstract verbs, and emotion words) and Group (2 levels). This revealed a significant (following Huynh–Feldt correction) interaction of Area, Word Category and Group: *F*(4, 136) = 4.833, p = .001. The left motor ROI, which arose as part of the cluster of activity with the Temporal ROIs but which was analysed separately, did not produce a significant main effect of Word Category (p = .09) or a Word Category interaction with Group (p = .866), though greatest activity for emotion words over other word types was seen in both groups.

The interaction of Area, Word Category and Group was unpacked in an analysis of each Area separately, employing an ANOVA with the factors Word Category (3 levels) and Group. Significant interactions between Word Category and Group were present only in Cingulate and Motor clusters (see Table 2, Fig. 2). Post-hoc t-tests of these interactions revealed significant between-group differences for emotion but not comparison word categories in both the Cingulate (*t*(34) = 2.116, p = .042) and Motor (*t*(34) = 2.867, p = .007) Areas. In fact, this hypoactivity for emotion words in the Cingulate Area correlated with the hypoactivity for emotion words seen in the Motor area (r = 863, p = .001), although no other correlations were seen between brain activity for word categories in other regions.

**Table 2:** Significant interactions in key regional clusters.

	Temporal cluster	Cingulate cluster	Motor cluster
Interactions			
(Word Category × Group)	N.S. (p > .9)	*F* (2, 68) = 3.353, ε = .839,	*F* (2, 68) = 3.364, p = .015
		p = .041	
Bilateral interactions	N.S. (p > .7)	*F* (2, 68) = 4.468, ε = .860,	*F* (2, 68) = 3.364, p = .04
(Word Category × Group)		p = .045	

Table 2: Significant interactions between Word Category and Group for the three regional clusters found active during word processing (left temporal, left cingulate and right motor cortex) are presented on the upper row. The results from a bilateral analysis computed for these same clusters together with the homotopic clusters in the contralateral hemisphere are reported on the bottom row. Values given are Huynh–Feldt corrected where appropriate.

A bilateral analysis using homologues of the ROIs collapsed in these three clusters confirmed this pattern of hypoactivity, producing an interaction of Area, Word Category and Group which was significant following Huynh–Feldt correction (*F*(4, 136) = 3.993, p = .004). Again, post-hoc analysis revealed that emotion words were the only word category to differ significantly between groups in the bilateral Cingulate (*t*(34) = 2.145, p = .039) and Motor (*t*(34) = 2.826, p = .008) Areas.

Within group analysis of all three major clusters together showed no significant word category differences for ASC, but several in the control group (*F*[2, 34] = 3.444, ε = .906, p = .043). These were significant in the Motor Area (F [2, 34] = 8.049, ε = 1.000, p = .001), where greater activity was evoked by emotion words than by animal names (*t*(17) = 3.413, p = .003) or abstract verbs (*t*(17) = 3.002, p = .008) respectively.

Correlations between bilateral activity for each word category in the three key clusters and the AQ were explored in order to assess a potential behavioural link with the hypoactivity for emotion words described above. Following the removal of two outliers (see Methods for detail), a highly significant correlation between activity elicited by emotion words in the Motor Area bilaterally and AQ score (r = − .679, p = .004) was seen in the ASC group. This correlation reflected that higher scores, indicating a greater number of autistic traits, were associated with lower activity to emotion words in the Motor Areas (see Supplementary materials, item S4). It remained significant following Bonferroni correction (to a corrected p-value of 0.0056, based on 9 tests) and confirmation with a non-parametric test (Spearman's Rho: r = − .677, p = .004). We did not find any significant correlations between AQ scores and activity for any of the other categories in the motor Area, and no significant correlations between AQ scores and activity for any word category in the cingulate or temporal Areas.

## Discussion

Brain activation elicited during passive reading of abstract emotion words and two comparison control categories was compared in ASC and typically-developing (TD) control participants. Whole-brain analyses of activity evoked by emotion words showed significantly reduced neurometabolic responses in ASC, specifically in bilateral motor areas and in the left insula and basal ganglia. ROI analysis confirmed and extended these findings. A statistically-significant interaction of the factors Area (3 levels: temporal, cingulate, motor), Word category (3) and group (2) revealed that in cingulate and motor areas (the former including BA 23, BA 32, the latter primary motor and premotor cortex [BA 4 and 6 respectively]), people with ASC showed significantly reduced activity than controls for emotion words, but not for abstract verbs and animal names. Reduced activity in the ASC group for emotion words did not appear in temporal cortex but only specifically in the motor and insular regions where the same TD controls showed the strongest emotion word-evoked activity (Moseley et al., 2012). Furthermore, motor systems activation in people with ASC significantly correlated with their number of autistic traits, as assessed by AQ scores.

### Action binds words to emotional meaning

Alongside elevated rates of alexithymia (Lombardo et al., 2007, Hill et al., 2004) and general deficits in emotion processing and recognition (Uljarevic and Hamilton, 2013), people with ASC show difficulty in the processing of mental or emotional states and words semantically related to emotions (Hobson and Lee, 1989, Baron‐Cohen et al., 1986, Baron-Cohen et al., 1994, Capps et al., 1992, Tager‐Flusberg, 1992, Happé, 1994, Tager-Flusberg and Sullivan, 1994, Tager-Flusberg and Sullivan, 1995, Jolliffe and Baron-Cohen, 1999). As these impairments must reflect a neurological correlate, we hypothesised that substantial differences might exist in how these individuals process and store words with emotional meaning.

Against a background of extensive atypical limbic structure and function in ASC (see Introduction), reduced activity here for emotion-related words is not in itself a surprising finding. Due to the poor temporal resolution of fMRI, it is not possible to determine whether limbic hypo-activation to emotion words reflects general problems with late stage, conscious *post-understanding* processing of emotion words or a deactivation of these emotion concepts in the earliest time window when semantic differences are reflected by brain response (Pulvermüller et al., 2009, Moseley et al., 2013b). Reduced activity in the limbic system was however accompanied by and correlated with reduced activity in the motor system, which in turn reflected the degree of autistic traits in the ASC group. On the basis of this data, we would venture to suggest that the under-activation in limbic systems and its accompanying motor hypoactivity are a hallmark of autism rather than an epiphenomenon.

Cortical language and motor systems are tightly entwined, such that passive language perception activates motor regions in a general fashion, as if simulating the spoken or written language (Fadiga et al., 2002, Floel et al., 2003, Meister et al., 2003). Over and above this, however, semantic theory and experimental data suggest that the meanings of both action words (Pulvermüller et al., 2001, Hauk et al., 2004, Hauk et al., 2008, Aziz-Zadeh and Damasio, 2008, Kemmerer et al., 2008, Boulenger et al., 2009, Shebani and Pulvermüller, 2013; see Pulvermüller and Fadiga, 2010, for review) *and* emotion words (Moseley et al., 2012) are ‘embodied’ in cortical motor systems — such that words do not activate these areas equally. Action words are represented somatotopically such that they activate the cortical motor regions for the effectors involved in that action. For emotion words, the link between emotions and the symbols (words) denoting them may be established by way of actions, and motor activation in emotion word processing may therefore index this link. Cortical motor systems are the vehicle of emotion expression. Because they refer to abstract concepts such as “fear” which cannot be easily pointed to or labelled, the meaning of emotion words is established by the use of the words in action contexts — facial expressions and emotional behaviours such as crying, screaming and gesticulating. As the only visible ‘signals’ of internal emotional states, semantic theory postulates that these actions are critical for bridging the gap between word and meaning and so, in the typical population, become incorporated in the neural network representing the meaning of these emotion terms (Moseley et al., 2012). During this process, the word comes to be associated with the internal feeling it describes, and so, in addition, these words possess ‘limbic tails’ (Pulvermüller and Schumann, 1994), activating neurons in limbic structures involved in processing the specific emotions denoted by these words. In the typically-developing participants, these areas included the orbitofrontal and frontopolar cortex, anterior cingulate gyrus, insula, and basal ganglia including putamen, caudate, and globus pallidum (Moseley et al., 2012; areas also highlighted in abstract word processing, see Vigliocco et al., 2013). In the motor system, these participants showed the greatest activity in motor systems for emotion words and the lowest for animal names with little or no semantic relationship to actions. Though abstract words, too, may be partially grounded in a multitude of sensorimotor action contexts (Wittgenstein, 1953, Barsalou, 1999, Lakoff and Johnson, 1980, Lakoff and Núñez, 2000, Gallese and Lakoff, 2005, Casasanto, 2009, Guan et al., 2013), they are detached from *specific* action schemas (Kiefer and Pulvermüller, 2012, Pulvermüller, 2013). As such, despite equal ratings in action-relatedness, they evoked less activity in cortical motor systems than did emotion words, and the hierarchy of motor activation in our typical population – highest for emotion words, lowest for animal names as according with their action-relatedness – sits comfortably within the previous literature (Pulvermüller and Fadiga, 2010).

In comparison to the typically-developing participants, individuals with ASC here exhibited reduced activity for emotion words in limbic and motor regions (primary motor and premotor cortex, the insula, cingulate and caudate nucleus: see Table 1). That reduced motor and limbic activity was restricted to emotion words, but not other word types, is evidence for the atypical alteration of the action-semantic link (via behaviours involving motor systems) between emotion words and their related emotions. Stringent matching of our word categories, particularly the abstract verbs, allowed us to refute the possibility that participants with ASC might have a particular difficulty with *all* verbs, or with all words of an abstract nature. The cause of the group difference in activity for emotion words can therefore be presumed to be related to the emotional and mental state content of emotion words (e.g. “dread”) that was absent in the case of equally abstract verbs such as “dwell”, revealing a genuine category-specific semantic abnormality.

### The role of motor systems in emotion word processing, alexithymia and ASC

The pattern of activity in the control group, who in a previous analysis showed substantial overlap in the motor activity evoked by abstract emotion words such as “fear” and that evoked by overt action words such as “frown”, is consistent with the theoretical grounding of emotional concepts via actions (Moseley et al., 2012). The precise role of such motor activity for emotional words, however, is as yet unclear.

Semantic theory postulates that words are represented in distributed “action–perception circuits” which link the representation of phonology and articulatory word features in core perisylvian language cortices with the representation of meaning in sensorimotor systems (Pulvermüller and Fadiga, 2010). Functional importance of these sensorimotor systems for the processing of action words is supported by a plethora of empirical work, which demonstrates a common neural substrate for movement and the understanding of action-related language (Fischer and Zwaan, 2008, Rueschemeyer et al., 2009). Among the strongest evidence is that relating deficits in action word processing to damage or disease of the motor system (Bak et al., 2001, Bak et al., 2006, Neininger and Pulvermueller, 2001, Neininger and Pulvermüller, 2003, Bak and Hodges, 2004, Cotelli et al., 2006, Grossman et al., 2008, Bak and Chandran, 2012, Kemmerer et al., 2012). The bidirectional influence between motor systems and action-semantic processing is such that differences were observed in the same patients with Parkinson's disease before and after dopaminergic treatment (Boulenger et al., 2008). Of the greatest relevance to the present dataset is our previous research, which demonstrated abnormalities and a deficit in action word processing in individuals with autism. A significant correlation was seen between hypoactivity for action words in motor cortex and slowness in a task of semantic processing: as activity in motor systems decreased in participants with ASC, reaction times for action word processing increased (Moseley et al., 2013a). This was a very course-grained task, simply asking participants to categorise words as actions or objects, which may be why this impairment did not manifest in significantly greater errors. The fact that autistic participants were nonetheless significantly impeded in this very simple task suggests, however, that they might be substantially impaired in more complex or subtle action word processing tasks, as were the patients described above. The motor hypoactivity for action words that we had observed with fMRI was further corroborated with temporally precise combined EEG-MEG recording of autistic participants (Moseley et al., 2014).

As we did not conduct a behavioural test of emotion word processing, we cannot here show a relationship between the hypoactivity seen in motor and limbic regions and emotion word processing errors or impairment. We would hypothesise that, as for action words, this activation plays a functional role in their processing — and would accordingly predict autistic participants to show category-specific deficits for emotion words when these are compared with other well-matched word stimuli. Modulation of motor systems certainly affects processing of emotion-related language (Glenberg et al., 2005), such that lesions or disorder of these areas would be expected to produce impairments.

Though we did not conduct our own behavioural test of word processing, the motor and limbic hypoactivity seen specifically for emotion words in this analysis is consistent with other autism research reporting difficulties understanding and using emotion words, and with general emotion processing impairments. At the neural level, it is consistent with the atypical structural features in limbic regions and primary motor cortex in ASC (Mostofsky et al., 2007), and with movement impairments in autism (Fournier et al., 2010). In order to investigate a functional connection between motor and limbic systems activity and emotion word processing, and thus link the present data with reported impairments, future research should investigate the overlap of movement impairment and alexithymia in ASC and directly test the correlation between hypoactivity and category-specific deficits in active behavioural tasks involving processing words related to emotions. In such an investigation, it would also be optimal to measure alexithymia directly by means of a specialised scale. We attempted to retrospectively collect data using the Toronto Alexithymia Scale (TAS-20: Bagby et al., 1994) after our study finished, but unfortunately had few respondents from our sample, such that the hypothesised relationship between motor/limbic hypoactivity and alexithymia itself (as opposed to the autism measure included in this study) remains tentative. Interestingly, the present study observed a significant correlation, whilst reading emotion words, between hypoactivity in the motor system and hypoactivity in the cingulate cortex (though not with activity in other regions examined, ruling out any general hypoactivity). Although causal primacy of emotion expression deficits (associated with atypical motor circuits) over emotion processing/recognition deficits (associated with atypical limbic cortices) cannot be supported by this data, the correlation between motor and limbic hypoactivity observed here does imply a relationship between the capacity to express one's emotions and the capacity to process and understand the emotions of the self and others, as implied in the idea of the self as proxy for emotion processing (Lombardo and Baron-Cohen, 2011).

Examination of ASC through the prism of movement disorder was considered by Leary and Hill (1996) but has been overshadowed by studies of the more prominent social-communication symptoms. More recently, however, atypical sensorimotor circuits have been argued to have wider implications for ASC, as they are suggested to contribute in a simulative manner to mentalising (Lombardo et al., 2010) and other aspects of social cognition such as empathy (Minio-Paluello et al., 2009). Alexithymia is just one indication of impairments in self-referential processing (Lombardo and Baron-Cohen, 2011), and the idea that the self is used as a proxy suggests that this impairment may be associated with the broader meta-representational deficit in autism. If conceptual understanding of emotion concepts requires the involvement of motor systems then these broad impairments may be a downstream reflection of fundamental abnormalities in motor circuits. Other social-communication impairments in ASC might similarly arise from motor dysfunction, given the relationship between motor, social and language development in childhood (Lenneberg, 1967, Iverson, 2010) and the early emergence of motor abnormalities prior to other features of ASC (Teitelbaum et al., 1998, Young et al., 2003, Esposito et al., 2009, Rogers, 2009). In ASC, correlations have been noted between movement difficulties and impaired social-communication skills (Mostofsky and Ewen, 2011). In addition, motor impairment predicts childhood and adolescent speech fluency and delay in both children with autism and their high-risk siblings (Gernsbacher et al., 2008, Bhat et al., 2012). In a previous publication, we demonstrated an association between motor hypoactivity and a category-specific impairment for action words (Moseley et al., 2013a). This study also found a correlation between motor hypoactivity and AQ scores, a notable finding given that the AQ largely measures the social difficulties and narrow interests in ASC and makes no reference to motor symptoms which would be naturally expected to correlate with motor hypoactivity. Moseley et al.'s (2013a) study fits with the perspective that brain circuits involving motor systems may underpin many higher cognitive processes.

Given this relationship between motor abnormality and ASC, the present work investigated the correlation between activity in motor areas evoked by emotion words and autistic traits. A highly significant correlation was seen between AQ scores in the ASC group and activity evoked by emotion words in the motor system bilaterally. This finding appeared following removal of two outliers, so further investigation with greater numbers is important to confirm this. It may be that impairment in emotion expression (dependent on the integrity of motor circuits controlling emotional behaviours) is a hallmark of autism and precedes and underlies the development of other symptoms, a perspective consistent with the aforementioned developmental primacy of motor symptoms of ASC. This suggestion requires further investigation in a non-correlational approach for statements of causal primacy to be justified.

Consistent with the previous finding related to action word processing (Moseley et al., 2013a), the current data demonstrates a relationship between autistic traits and motor system hypoactivity during processing of *another* word-type associated with motor systems (Moseley et al., 2012). As motor systems are typically involved in processing both action words and emotion words, the hypoactivity seen here is symptomatic of underlying abnormalities in motor circuits (and/or their connections) that correlate with ASC traits. It suggests a relationship between disorder of sensorimotor systems (and/or their connections) and the emotional-affective and socio-communicative difficulties seen in ASC. Further research is clearly necessary to elucidate a putative link between motor systems abnormality, emotion word processing deficits, and other socio-communicative symptoms of ASC. The role of motor systems in higher cognitive processes is increasingly recognised in neuroscience (Jeannerod, 2006, Fischer and Zwaan, 2008, Pulvermüller and Fadiga, 2010, Rizzolatti and Sinigaglia, 2010), and given the relationship between motor, cognitive and emotional processing, further study of motor impairments and their role in ASC may be timely and fruitful for interventions.

## Conclusion

Using event-related fMRI, we explored the representation of emotion words in comparison to abstract verbs and animal names in the typical population and people with ASC. Our data revealed substantial differences between participant groups at a whole-brain level: the control group showed significantly greater activity for emotion words in motor cortex and limbic regions such as the cingulate and the basal ganglia, all of which have previously been implicated in emotion and emotion word processing. In addition, more thorough ROI analysis of temporal, cingulate and motor regions revealed a category-specific reduction for emotion words in the cingulate cortex and the motor system — both regions previously implicated in emotion word processing. This hypoactivation is consistent with ASC deficits in emotion expression and mentalising outside the language domain. We suggest this may constitute an underlying neural substrate for impairments in emotion word processing, which seems to rely on these motor and limbic cortical areas. We also report a significant correlation between motor hypoactivity in ASC and the expression of autistic traits.

## Funding

This work was supported by the 10.13039/501100000265Medical Research Council (MRC) (MC_US_A060_0034, U1055.04.003.00001.01 to F.P., MC_US_A060_0043, MC-A060-5PQ90 to Y. S., MRC studentship to R.M.).

## Appendix A Supplementary data

Supplementary material. **Table S1:** Psycholinguistic and semantic properties of animal names, abstract verbs and emotion words. **Table S2:** Experimental word stimuli. **Table S3:** MNI coordinates for between-groups contrasts (controls > ASC) for abstract verbs and animal names. **Supplementary image, S4:** scatterplot of correlation analysis.

## References

[bb0005] Adolphs R., Damasio H., Tranel D., Cooper G., Damasio A.R. (2000). A role for somatosensory cortices in the visual recognition of emotion as revealed by three-dimensional lesion mapping. J. Neurosci..

[bb0010] Aylward E.H., Minshew N.J., Goldstein G., Honeycutt N.A., Augustine A.M., Yates K.O., Pearlson G.D. (1999). MRI volumes of amygdala and hippocampus in non-mentally retarded autistic adolescents and adults. Neurology.

[bb0015] Aziz-Zadeh L., Damasio A. (2008). Embodied semantics for actions: findings from functional brain imaging. J. Physiol. Paris.

[bb0020] Bagby R.M., Parker J.D., Taylor G.J. (1994). The twenty-item Toronto Alexithymia Scale—I. Item selection and cross-validation of the factor structure.. J. Psychosom. Res..

[bb0025] Bak T.H., Chandran S. (2012). What wires together dies together: verbs, actions and neurodegeneration in motor neuron disease. Cortex.

[bb0030] Bak T.H., Hodges J.R. (2004). The effects of motor neurone disease on language: further evidence. Brain Lang..

[bb0035] Bak T.H., O'Donovan D.G., Xuereb J.H., Boniface S., Hodges J.R. (2001). Selective impairment of verb processing associated with pathological changes in Brodmann areas 44 and 45 in the motor neurone disease—dementia–aphasia syndrome. Brain.

[bb0040] Bak T.H., Yancopoulou D., Nestor P.J., Xuereb J.H., Spillantini M.G., Pulvermüller F., Hodges J.R. (2006). Clinical, imaging and pathological correlates of a hereditary deficit in verb and action processing. Brain.

[bb0045] Barnea-Goraly N., Kwon H., Menon V., Eliez S., Lotspeich L., Reiss A.L. (2004). White matter structure in autism: preliminary evidence from diffusion tensor imaging. Biol. Psychiatry.

[bb0050] Baron-Cohen S. (1995).

[bb0060] Baron-Cohen S., Ring H., Moriarty J., Schmitz B., Costa D.U.R.V.A.L., Ell P.E.T.E.R. (1994). Recognition of mental state terms. Clinical findings in children with autism and a functional neuroimaging study of normal adults. Br. J. Psychiatry.

[bb0070] Baron-Cohen S., Wheelwright S., Jolliffe A.T. (1997). Is there a“ language of the eyes”? Evidence from normal adults, and adults with autism or Asperger syndrome. Vis. Cogn..

[bb0075] Baron-Cohen S., Wheelwright S., Skinner R., Martin J., Clubley E. (2001). The autism-spectrum quotient (AQ): evidence from asperger syndrome/high-functioning autism, males and females, scientists and mathematicians. J. Autism Dev. Disord..

[bb0055] Baron‐Cohen S., Leslie A.M., Frith U. (1986). Mechanical, behavioural and intentional understanding of picture stories in autistic children. Br. J. Dev. Psychol..

[bb0065] Baron‐Cohen S., Wheelwright S., Hill J., Raste Y., Plumb I. (2001). The “Reading the Mind in the Eyes” test revised version: a study with normal adults, and adults with Asperger syndrome or high‐functioning autism. J. Child Psychol. Psychiatry.

[bb0080] Barsalou L.W. (1999). Perceptual symbol systems. Behav. Brain Sci..

[bb0085] Barsalou L.W. (2008). Grounded cognition. Annu. Rev. Psychol..

[bb0090] Bauman M.L., Kemper T.L., Bauman M.L., Kemper T.L. (1994). The Neurobiology of Autism.

[bb0095] Beall P.M., Moody E.J., McIntosh D.N., Hepburn S.L., Reed C.L. (2008). Rapid facial reactions to emotional facial expressions in typically developing children and children with autism spectrum disorder. J. Exp. Child Psychol..

[bb0100] Ben Shalom D., Mostofsky S.H., Hazlett R.L., Goldberg M.C., Landa R.J., Faran Y., Hoehn-Saric R. (2006). Normal physiological emotions but differences in expression of conscious feelings in children with high-functioning autism. J. Autism Dev. Disord..

[bb0105] Bhat A.N., Galloway J.C., Landa R.J. (2012). Relation between early motor delay and later communication delay in infants at risk for autism. Infant Behav. Dev..

[bb0110] Bölte S., Feineis-Matthews S., Poustka F. (2008). Brief report: emotional processing in high-functioning autism—physiological reactivity and affective report. J. Autism Dev. Disord..

[bb0115] Bonilha L., Cendes F., Rorden C., Eckert M., Dalgalarrondo P., Li L.M., Steiner C.E. (2008). Gray and white matter imbalance—typical structural abnormality underlying classic autism?. Brain and Development.

[bb0125] Boulenger V., Mechtouff L., Thobois S., Broussolle E., Jeannerod M., Nazir T.A. (2008). Word processing in Parkinson's disease is impaired for action verbs but not for concrete nouns. Neuropsychologia.

[bb0120] Boulenger V., Hauk O., Pulvermüller F. (2009). Grasping ideas with the motor system: semantic somatotopy in idiom comprehension. Cereb. Cortex.

[bb0130] Bradley M.M., Lang P.J. (1994). Measuring emotion: the self-assessment manikin and the semantic differential. J. Behav. Ther. Exp. Psychol..

[bb0135] Braverman M., Fein D., Lucci D., Waterhouse L. (1989). Affect comprehension in children with pervasive developmental disorders. J. Autism Dev. Disord..

[bb0140] Calder A.J., Lawrence A.D., Young A.W. (2001). Neuropsychology of fear and loathing. Nat. Rev. Neurosci..

[bb0145] Capps L., Yirmiya N., Sigman M. (1992). Understanding of simple and complex emotions in non‐retarded children with autism. J. Child Psychol. Psychiatry.

[bb9000] Capps L., Kasari C., Yirmiya N., Sigman M. (1993). Parental perception of emotional expressiveness in children with autism. J. Consult. Clin. Psychol..

[bb0150] Carroll J.B. (1993).

[bb0155] Casasanto D. (2009). Embodiment of abstract concepts: good and bad in right-and left-handers. J. Exp. Psychol. Gen..

[bb0160] Cattaneo L., Fabbri-Destro M., Boria S., Pieraccini C., Monti A., Cossu G., Rizzolatti G. (2007). Impairment of actions chains in autism and its possible role in intention understanding. Proc. Natl. Acad. Sci..

[bb0165] Cattell R.B. (1980). They talk of some strict testing of us—Pish. Behav. Brain Sci..

[bb0170] Cattell R.B., Cattell A.K.S. (1960).

[bb0175] Cheung C., Chua S.E., Cheung V., Khong P.L., Tai K.S., Wong T.K.W., McAlonan G.M. (2009). White matter fractional anisotrophy differences and correlates of diagnostic symptoms in autism. J. Child Psychol. Psychiatry.

[bb0185] Colom R., García-López O. (2002). Sex differences in fluid intelligence among high school graduates. Personal. Individ. Differ..

[bb0180] Colom R., Flores-Mendoza C., Rebollo I. (2003). Working memory and intelligence. Personal. Individ. Differ..

[bb9005] Cotelli M., Borroni B., Manenti R., Alberici A., Calabria M., Agosti C., Arévalo A., Ginex V., Ortelli P., Binetti G., Zanetti O., Padovani A., Cappa S.F. (2006). Action and object naming in frontotemporal dementia, progressive supranuclear palsy, and corticobasal degeneration. Neuropsychology.

[bb0190] Corona R., Dissanayake C., Arbelle S., Wellington P., Sigman M. (1998). Is affect aversive to young children with autism? Behavioral and cardiac responses to experimenter distress. Child Dev..

[bb0195] Dawson G., McKissick F.C. (1984). Self-recognition in autistic children. J. Autism Dev. Disord..

[bb0200] Duncan J., Seitz R.J., Kolodny J., Bor D., Herzog H., Ahmed A., Emslie H. (2000). A neural basis for general intelligence. Science.

[bb0205] Esposito G., Venuti P., Maestro S., Muratori F. (2009). An exploration of symmetry in early autism spectrum disorders: analysis of lying. Brain and Development.

[bb0210] Fadiga L., Craighero L., Buccino G., Rizzolatti G. (2002). Speech listening specifically modulates the excitability of tongue muscles: a TMS study. Eur. J. Neurosci..

[bb0220] Fischer M.H., Zwaan R.A. (2008). Embodied language: a review of the role of the motor system in language comprehension. Q. J. Exp. Psychol..

[bb0215] Floel A., Ellger T., Breitenstein C., Knecht S. (2003). Language perception activates the hand motor cortex: implications for motor theories of speech perception. Eur. J. Neurosci..

[bb0225] Fournier K.A., Hass C.J., Naik S.K., Lodha N., Cauraugh J.H. (2010). Motor coordination in autism spectrum disorders: a synthesis and meta-analysis. J. Autism Dev. Disord..

[bb0230] Gallese V., Lakoff G. (2005). The brain's concepts: the role of the sensory-motor system in conceptual knowledge. Cogn. Neuropsychol..

[bb0235] Gernsbacher M.A., Sauer E.A., Geye H.M., Schweigert E.K., Hill Goldsmith H. (2008). Infant and toddler oral‐and manual‐motor skills predict later speech fluency in autism. J. Child Psychol. Psychiatry.

[bb0245] Girgis R.R., Minshew N.J., Melhem N.M., Nutche J.J., Keshavan M.S., Hardan A.Y. (2007). Volumetric alterations of the orbitofrontal cortex in autism. Prog. Neuro-Psychopharmacol. Biol. Psychiatry.

[bb0240] Glenberg A.M., Havas D., Becker R., Rinck M., Pecher D., Zwaan R.A. (2005). Grounding Cognition: The Role of Perception and Action in Memory, Language and Thinking.

[bb0250] Golan O., Baron-Cohen S., Hill J.J., Rutherford M.D. (2007). The ‘reading the mind in the voice’ test-revised: a study of complex emotion recognition in adults with and without autism spectrum conditions. J. Autism Dev. Disord..

[bb9010] Grossman M., Anderson C., Khan A., Avants B., Elman L., McCluskey L. (2008). Impaired action knowledge in amyotrophic lateral sclerosis. Neurology.

[bb0260] Guan C.Q., Meng W., Yao R., Glenberg A.M. (2013). The motor system contributes to comprehension of abstract language. PLoS ONE.

[bb0265] Gustafsson J.E. (1984). A unifying model for the structure of intellectual abilities. Intelligence.

[bb0270] Happé F.G. (1994). An advanced test of theory of mind: understanding of story characters' thoughts and feelings by able autistic, mentally handicapped, and normal children and adults. J. Autism Dev. Disord..

[bb0275] Hauk O., Johnsrude I., Pulvermüller F. (2004). Somatotopic representation of action words in human motor and premotor cortex. Neuron.

[bb0280] Hauk O., Shtyrov Y., Pulvermüller F. (2008). The time course of action and action-word comprehension in the human brain as revealed by neurophysiology. J. Physiol. Paris.

[bb0285] Haznedar M.M., Buchsbaum M.S., Metzger M., Solimando A., Spiegel-Cohen J., Hollander E. (1997). Anterior cingulate gyrus volume and glucose metabolism in autistic disorder. Am. J. Psychiatr..

[bb0290] Haznedar M.M., Buchsbaum M.S., Wei T.C., Hof P.R., Cartwright C., Bienstock C.A., Hollander E. (2000). Limbic circuitry in patients with autism spectrum disorders studied with positron emission tomography and magnetic resonance imaging. Am. J. Psychiatr..

[bb0295] Hill E., Berthoz S., Frith U. (2004). Brief report: cognitive processing of own emotions in individuals with autistic spectrum disorder and in their relatives. J. Autism Dev. Disord..

[bb0300] Hobson R.P. (1986). The autistic child's appraisal of expressions of emotion. J. Child Psychol. Psychiatry.

[bb0305] Hobson R.P. (1986). The autistic child's appraisal of expressions of emotion: a further study. J. Child Psychol. Psychiatry.

[bb0310] Hobson R.P., Lee A. (1989). Emotion-related and abstract concepts in autistic people: evidence from the British Picture Vocabulary Scale. J. Autism Dev. Disord..

[bb0315] Hobson R.P., Chidambi G., Lee A., Meyer J., Müller U., Carpendale J.I.M., Racine T.P. (2006). Foundations for self-awareness: an exploration through autism. Monogr. Soc. Res. Child Dev..

[bb0320] Howard M.A., Cowell P.E., Boucher J., Broks P., Mayes A., Farrant A., Roberts N. (2000). Convergent neuroanatomical and behavioural evidence of an amygdala hypothesis of autism. Neuroreport.

[bb0325] Humphreys K., Minshew N., Leonard G.L., Behrmann M. (2007). A fine-grained analysis of facial expression processing in high-functioning adults with autism. Neuropsychologia.

[bb0330] Iverson J.M. (2010). Developing language in a developing body: the relationship between motor development and language development*. J. Child Lang..

[bb0335] Jeannerod M. (2006).

[bb0340] Jensen A. (1980). Précis of bias in mental testing. Behav. Brain Sci..

[bb0345] Jolliffe T., Baron-Cohen S. (1999). The strange stories test: a replication with high-functioning adults with autism or Asperger syndrome. J. Autism Dev. Disord..

[bb0350] Kanner L. (1943). Autistic disturbances of affective contact. Nerv. Child.

[bb0355] Kasari C., Sigman M., Yirmiya N., Mundy P., Kaiser A.P., Gray D.B. (1993). Enhancing Children's Communication: Research Foundation for Early Language Interventions.

[bb0360] Kemmerer D., Castillo J.G., Talavage T., Patterson S., Wiley C. (2008). Neuroanatomical distribution of five semantic components of verbs: evidence from fMRI. Brain Lang..

[bb0370] Kemmerer D., Rudrauf D., Manzel K., Tranel D. (2012). Behavioral patterns and lesion sites associated with impaired processing of lexical and conceptual knowledge of actions. Cortex.

[bb0365] Kiefer M., Pulvermüller F. (2012). Conceptual representations in mind and brain: theoretical developments, current evidence and future directions. Cortex.

[bb0375] Lakoff G., Johnson M. (1980).

[bb0380] Lakoff G., Núñez R. (2000).

[bb0385] Leary M.R., Hill D.A. (1996). Moving on: autism and movement disturbance. Ment. Retard..

[bb9015] Lenneberg E.H. (1967).

[bb0390] Leys C., Ley C., Klein O., Bernard P., Licata L. (2013). Detecting outliers: do not use standard deviation around the mean, use absolute deviation around the median. J. Exp. Soc. Psychol..

[bb0400] Lombardo M.V., Baron-Cohen S. (2011). The role of the self in mind blindness in autism. Conscious. Cogn..

[bb0395] Lombardo M.V., Barnes J.L., Wheelwright S.J., Baron-Cohen S. (2007). Self-referential cognition and empathy in autism. PLoS ONE.

[bb0405] Lombardo M.V., Chakrabarti B., Bullmore E.T., Wheelwright S.J., Sadek S.A., Suckling J., MRC AIMS Consortium (2010). Shared neural circuits for mentalizing about the self and others. J. Cogn. Neurosci..

[bb0410] McAlonan G.M., Cheung C., Cheung V., Wong N., Suckling J., Chua S.E. (2009). Differential effects on white-matter systems in high-functioning autism and Asperger's syndrome. Psychol. Med..

[bb0415] McIntosh D.N., Reichmann‐Decker A., Winkielman P., Wilbarger J.L. (2006). When the social mirror breaks: deficits in automatic, but not voluntary, mimicry of emotional facial expressions in autism. Dev. Sci..

[bb0420] Meister I.G., Boroojerdi B., Foltys H., Sparing R., Huber W., Topper R. (2003). Motor cortex hand area and speech: implications for the development of language. Neuropsychologia.

[bb0425] Minio-Paluello I., Baron-Cohen S., Avenanti A., Walsh V., Aglioti S.M. (2009). Absence of embodied empathy during pain observation in Asperger syndrome. Biol. Psychiatry.

[bb0430] Moseley R., Carota F., Hauk O., Mohr B., Pulvermüller F. (2012). A role for the motor system in binding abstract emotional meaning. Cereb. Cortex.

[bb0435] Moseley R.L., Mohr B., Lombardo M.V., Baron-Cohen S., Hauk O., Pulvermüller F. (2013). Brain and behavioral correlates of action semantic deficits in autism. Front. Hum. Neurosci..

[bb0440] Moseley R.L., Pulvermüller F., Shtyrov Y. (2013). Sensorimotor semantics on the spot: brain activity dissociates between conceptual categories within 150 ms. Sci. Rep..

[bb9020] Moseley R.L., Mohr B., Lombardo M.V., Baron-Cohen S., Pulvermüller F., Shtyrov Y. (2013). Brain routes for reading in adults with and without autism. J. Autism Dev. Disord..

[bb0450] Mostofsky S.H., Ewen J.B. (2011). Altered connectivity and action model formation in autism is autism. Neuroscientist.

[bb0445] Mostofsky S.H., Burgess M.P., Larson J.C.G. (2007). Increased motor cortex white matter volume predicts motor impairment in autism. Brain.

[bb0455] Neininger B., Pulvermueller F. (2001). The right hemisphere's role in action word processing: a double case study. Neurocase.

[bb0460] Neininger B., Pulvermüller F. (2003). Word-category specific deficits after lesions in the right hemisphere. Neuropsychologia.

[bb0465] Neuman C.J., Hill S.D. (1978). Self‐recognition and stimulus preference in autistic children. Dev. Psychobiol..

[bb0470] Oberman L.M., Winkielman P., Ramachandran V.S. (2009). Slow echo: facial EMG evidence for the delay of spontaneous, but not voluntary, emotional mimicry in children with autism spectrum disorders. Dev. Sci..

[bb0475] Ohnishi T., Matsuda H., Hashimoto T., Kunihiro T., Nishikawa M., Uema T., Sasaki M. (2000). Abnormal regional cerebral blood flow in childhood autism. Brain.

[bb0480] Osgood C., Suci G., Tannenhaus P. (1975).

[bb0485] Philip R.C.M., Whalley H.C., Stanfield A.C., Sprengelmeyer R., Santos I.M., Young A.W., Hall J. (2010). Deficits in facial, body movement and vocal emotional processing in autism spectrum disorders. Psychol. Med..

[bb0490] Pitcher D., Garrido L., Walsh V., Duchaine B.C. (2008). Transcranial magnetic stimulation disrupts the perception and embodiment of facial expressions. J. Neurosci..

[bb0495] Pugliese L., Catani M., Ameis S., Dell'Acqua F., de Schotten M.T., Murphy C., Murphy D.G. (2009). The anatomy of extended limbic pathways in Asperger syndrome: a preliminary diffusion tensor imaging tractography study. NeuroImage.

[bb0500] Pulvermüller F. (2012). Meaning and the brain: the neurosemantics of referential, interactive, and combinatorial knowledge. J. Neurolinguistics.

[bb0505] Pulvermüller F. (2013). How neurons make meaning: brain mechanisms for embodied and abstract-symbolic semantics. Trends Cogn. Sci..

[bb0510] Pulvermüller F., Fadiga L. (2010). Active perception: sensorimotor circuits as a cortical basis for language. Nat. Rev. Neurosci..

[bb0520] Pulvermüller F., Schumann J.H. (1994). Neurobiological mechanisms of language acquisition. Lang. Learn..

[bb0515] Pulvermüller F., Härle M., Hummel F. (2001). Walking or talking? Behavioral and neurophysiological correlates of action verb processing. Brain Lang..

[bb0525] Pulvermüller F., Shtyrov Y., Hauk O. (2009). Understanding in an instant: neurophysiological evidence for mechanistic language circuits in the brain. Brain Lang..

[bb9025] Pulvermüller F., Moseley R.L., Egorova N., Shebani Z., Boulenger V. (2014). Neuropsychologia.

[bb0530] Raymond G.V., Bauman M.L., Kemper T.L. (1995). Hippocampus in autism: a Golgi analysis. Acta Neuropathol..

[bb0535] Rizzolatti G., Fabbri-Destro M. (2010). Mirror neurons: from discovery to autism. Exp. Brain Res..

[bb0540] Rizzolatti G., Sinigaglia C. (2010). The functional role of the parieto-frontal mirror circuit: interpretations and misinterpretations. Nat. Rev. Neurosci..

[bb0545] Rogers S.J. (2009). What are infant siblings teaching us about autism in infancy?. Autism Res..

[bb0550] Rueschemeyer S.A., Lindemann O., Van Elk M., Bekkering H. (2009). Embodied cognition: the interplay between automatic resonance and selection‐for‐action mechanisms. Eur. J. Soc. Psychol..

[bb0555] Rutherford M.D., Baron-Cohen S., Wheelwright S. (2002). Reading the mind in the voice: a study with normal adults and adults with Asperger syndrome and high functioning autism. J. Autism Dev. Disord..

[bb0560] Salmond C.H., De Haan M., Friston K.J., Gadian D.G., Vargha-Khadem F. (2003). Investigating individual differences in brain abnormalities in autism. Philos. Trans. R. Soc. Lond. Ser. B Biol. Sci..

[bb0565] Schumann C.M., Amaral D.G. (2006). Stereological analysis of amygdala neuron number in autism. J. Neurosci..

[bb0570] Schumann C.M., Hamstra J., Goodlin-Jones B.L., Lotspeich L.J., Kwon H., Buonocore M.H., Amaral D.G. (2004). The amygdala is enlarged in children but not adolescents with autism; the hippocampus is enlarged at all ages. J. Neurosci..

[bb0575] Shebani Z., Pulvermüller F. (2013). Moving the hands and feet specifically impairs working memory for arm-and leg-related action words. Cortex.

[bb0580] Sigman M.D., Kasari C., Kwon J.H., Yirmiya N. (1992). Responses to the negative emotions of others by autistic, mentally retarded, and normal children. Child Dev..

[bb0585] Silani G., Bird G., Brindley R., Singer T., Frith C., Frith U. (2008). Levels of emotional awareness and autism: an fMRI study. Soc. Neurosci..

[bb0590] Snow M.E., Hertzig M.E., Shapiro T. (1987). Expression of emotion in young autistic children. J. Am. Acad. Child Adolesc. Psychiatry.

[bb0595] Szatmari P., Georgiades S., Duku E., Zwaigenbaum L., Goldberg J., Bennett T. (2008). Alexithymia in parents of children with autism spectrum disorder. J. Autism Dev. Disord..

[bb0600] Tager‐Flusberg H. (1992). Autistic children's talk about psychological states: deficits in the early acquisition of a theory of mind. Child Dev..

[bb0605] Tager-Flusberg H., Sullivan K. (1994). A second look at second-order belief attribution in autism. J. Autism Dev. Disord..

[bb0610] Tager-Flusberg H., Sullivan K. (1995). Attributing mental states to story characters: a comparison of narratives produced by autistic and mentally retarded individuals. Appl. Psycholinguist..

[bb0615] Teitelbaum P., Teitelbaum O., Nye J., Fryman J., Maurer R.G. (1998). Movement analysis in infancy may be useful for early diagnosis of autism. Proc. Natl. Acad. Sci..

[bb0620] Uddin L.Q., Menon V. (2009). The anterior insula in autism: under-connected and under-examined. Neurosci. Biobehav. Rev..

[bb0625] Uljarevic M., Hamilton A. (2013). Recognition of emotions in autism: a formal meta-analysis. J. Autism Dev. Disord..

[bb0630] Vigliocco G., Kousta S.T., Della Rosa P.A., Vinson D.P., Tettamanti M., Devlin J.T., Cappa S.F. (2013). The neural representation of abstract words: the role of emotion. Cereb. Cortex.

[bb9030] Wechsler D. (1944).

[bb0635] White S., Hill E., Happé F., Frith U. (2009). Revisiting the strange stories: revealing mentalizing impairments in autism. Child Dev..

[bb0640] Williams J.H., Whiten A., Suddendorf T., Perrett D.I. (2001). Imitation, mirror neurons and autism. Neurosci. Biobehav. Rev..

[bb0645] Wittgenstein L. (1953).

[bb0650] Woodbury-Smith M.R., Robinson J., Wheelwright S., Baron-Cohen S. (2005). Screening adults for Asperger syndrome using the AQ: a preliminary study of its diagnostic validity in clinical practice. J. Autism Dev. Disord..

[bb0655] Yirmiya N., Kasari C., Sigman M., Mundy P. (1989). Facial expressions of affect in autistic, mentally retarded and normal children. J. Child Psychol. Psychiatry.

[bb0660] Young R.L., Brewer N., Pattison C. (2003). Parental identification of early behavioural abnormalities in children with autistic disorder. Autism.

