# Supplementary material for: Lost for emotion words: What motor and limbic brain activity reveals about autism and semantic theory
Source: Neuroimage. 2015 Jan 1;104:413–22. doi: 10.1016/j.neuroimage.2014.09.046 (PMC4265725; doi:10.1016/j.neuroimage.2014.09.046)
Supplement: Supplementary file 1 — Supplementary material. Table S1: Psycholinguistic and semantic properties of animal names, abstract verbs and emotion words. Table S2: Experimental word stimuli. Table S3: MNI coordinates for between-groups contrasts (controls > ASC) for abstract verbs and animal names. Supplementary image, S4: scatterplot of correlation analysis. [file mmc1.doc]

# Supplementary Materials

**Table S1:** Psycholinguistic and semantic properties of animal names, abstract verbs and emotion words.

**Table S2:** Experimental word stimuli.

**Table S3:** MNI coordinates for between-groups contrasts (controls > ASC) for abstract verbs and animal names.

**Supplementary image, S4**: scatterplot of correlation analysis.

**Table S1:**

|  | *Animal names* | *Abstract verbs* | *Emotion words* | **Main effect of**  **word-type (F)** |
| --- | --- | --- | --- | --- |
| Length | 4.28 (.68) | 4.60 (.63) | 4.5 (.761) | 2.37 (p = .112) |
| Bigram freq.  Trigram freq.  Word frequency | 34618.40 (17581.04)  4454.73 (3146.06)  9.15 (23.46) | 41159.19 (18143.02)  4503.48 (4630.32)  5.88 (11.49) | 41405.62 (15736.88)  3340.77 (2522.75)  7.00 (15.80) | 1.73 (p = .162)  .750 (p = .453)  2.97 (p = .517) |
| No. of neighbours | 8.75 (5.97) | 6.40 (5.46) | 6.45 (5.15) | 2.077 (p = .099) |
| Imageability | 6.30 (.68) | 2.28 (1.02) | 2.24 (1.07) | 235.79 (p =.001) |
| Concreteness  Action-relatedness | 6.59 (.36)  1.63 (.82) | 2.61 (.69)  4.16 (1.27) | 2.72 (.92)  4.17 (1.30) | 454.92 (p =.001)  61.828 (p =.001) |
| Valence  Arousal | 3.71 (.37)  1.27 (.58) | 3.64 (1.09)  2.04 (1.01) | 2.07 (.47)  1.80 (1.02) | 36.405 (p =.001)  8.149 (p =.001) |
|  |  |  |  |  |

Table S1: Psycholinguistic and semantic word properties of experimental word categories. Means and standard errors (brackets) are reported for each word category, along with results of an ANOVA (animal names x abstract verbs x abstract emotion words). Bigram and trigram frequencies are taken from the CELEX database, number of neighbours from the MRC Psycholinguistic Database, and semantic values from an independent semantic rating study.

**Table S2: Experimental word stimuli**

| **Animal names** | **Abstract verbs** | **Emotion words** |
| --- | --- | --- |
| sloth  snail  snake  pike  skunk  gull  clam  hawk  hare  mouse  crab  trout  sheep  bear  bull  whale  moose  goat  goose  duck  hen  shrimp  wasp  frog  shark  dove  vole  cow  mole  fox  crow  moth  slug  pig  seal  squid  quail  deer  toad  worm | skimp  glut  bilk  stash  coax  snare  heal  cite  glean  parse  soothe  dwell  pall  fend  fetch  waive  faze  soak  lapse  drift  slake  trust  shirk  feign  lure  stun  shine  botch  strive  bid  dare  quell  bate  cease  lodge  tempt  fade  reap  taint  bide | hate  dread  rile  huff  mope  chafe  peeve  scare  irk  miff  ail  spite  gripe  grouse  daunt  sulk  mock  gloat  spurn  gibe |

Table S2: Experimental word stimuli displayed in lowercase during fMRI scanning.

**Table S3: MNI coordinates for CONTROLS > ASC contrasts**

|  | *x y z* | *Cluster size* | *P (uncorr. .001)* |
| --- | --- | --- | --- |
| **Abstract verbs**  L. BA 6  L. BA 6  L. BA 6  L. Fusiform gyrus (BA 37)  L. BA 19  L. Fusiform gyrus (BA 37)  L. Pars triangularis (BA 45)  L. Cingulate gyrus (BA 32)  L. BA 6  L. Insula (BA 48)  L. BA 18  R. BA 17  L. Superior temporal gyrus (BA 22)  L. Middle temporal gyrus (BA 21)  R. BA 46  R. BA 46  R. BA 46  **Animal names**  L. insula (BA 48)  L. pars orbitalis (BA 47)  L. pars triangularis (BA 45)  R. BA 46  R. BA 46  L. fusiform gyrus (BA 37)  L. fusiform gyrus (BA 37)  L. fusiform gyrus (BA 37)  L. superior temporal (BA 22)  L. Pars orbitalis (BA 47)  L. orbitofrontal (BA 11)  R. BA 25  R. BA 25  Subcortical  Subcortical  L. orbitofrontal (BA 11) | -56, 6, 20  -54, 2, 40  -42, -8, 50  -42, -40, -16  -46, -74, -10  -42, -50, -26  -52, 36, 2  -14, 38, 18  -6, 0, 64  -62, -18, 18  -8, -84, 20  2, -74, 10  -60, -36, 2  -52, -28 -2  38, 54, 18  32, 44, 20  22, 50, 16  -42, 26, 18  -32, 18, 0  -46, 38, 0  36, 50, 18  28, 44, 18  -42, -48, -16  -38, -44, -24  -38, -34, -20  -60, -38, 2  -30, 32, -16  -20, 30, -16  14, 14, 2  6, 4, -2  -18, 10, 6  -10, 4, 6  -18, 14, -4 | 746  358  67  76  164  77  126  118  94  1900  205  332  136  109  106  98 | .001  .001  .049  .087  .017  .085  .032  .038  .060  .001  .009  .001  .027  .045  .048  .056 |

Table S3: MNI coordinates generated for comparisons of the control group with the ASC group for abstract verbs and animal names. Coordinates therefore reflect areas of significantly greater activity in the control than the ASC group, reported at a cluster-level significance threshold of p < .001 (uncorrected). None survived FWE-correction.

**Supplementary image, S4: Correlations between AQ scores and activity evoked by emotion words in the bilateral motor Area.**


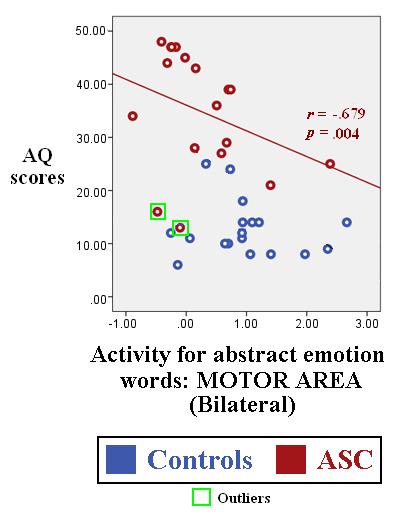


Image S4: Scatter-graph for the significant correlation of AQ scores in the ASC group and activity evoked by emotion words in the motor Area bilaterally. ASC participants are shown in red. Correlations were not statistically examined for the control group, given that they were not the focus of our hypotheses: these scores (blue) are displayed just for comparative purposes. Two outliers (see Methods) are identified in green.
